# A sequence-based classifier distinguishes phenotype-associated genes from other gene models in plants

**DOI:** 10.1101/gr.281802.125

**Published:** 2026-09

**Authors:** Nikee Shrestha, Zhongjie Ji, Xiuru Dai, Pinghua Li, James C. Schnable

**Affiliations:** 1 Center for Plant Science Innovation, University of Nebraska–Lincoln, Lincoln, Nebraska 68503, USA;; 2 Department of Agronomy and Horticulture, University of Nebraska–Lincoln, Lincoln, Nebraska 68583, USA;; 3 State Key Laboratory of Crop Biology, College of Agronomic Sciences, Shandong Agricultural University, Tai'an, Shandong 271018, China

## Abstract

Only a small fraction of annotated plant genes possess experimentally validated associations with specific phenotypes. Phenotype-associated genes have distinct structural, molecular, and evolutionary characteristics compared with nonvalidated gene models. Here, we develop a simple classifier that uses sequence and evolutionary features, which can be generated for any species with an annotated reference genome assembly, to accurately distinguish phenotype-associated genes from both the overall population of annotated gene models and a specific set of genes identified as being tolerant of premature stop mutations. A model trained solely on genes from maize (*Zea mays*) identifies and prioritizes rice (*Oryza sativa*) and *Arabidopsis* (*Arabidopsis thaliana*) genes that are highly enriched in genes with experimentally validated links to phenotypes in both of these evolutionarily distant species. Gene models predicted to have a higher probability of being linked to phenotypes display patterns consistent with known biological properties of phenotype-associated genes. Notably, the sets of genes predicted to have a high probability of being linked to phenotype variation do not consist exclusively of well-characterized gene families but included many uncharacterized gene families carrying domains of unknown function. The quantitative scores generated by this model offer a valuable resource for prioritizing and exploring the vast number of uncharacterized gene models in plants, reducing the risk of failure in future reverse genetic efforts and potentially accelerating gene discovery and functional annotation in crops.

The genomes of typical multicellular eukaryotes contain tens of thousands of gene model annotations. However, few of these genes have been the subject of specific genetic or molecular investigations. Indeed, in *Arabidopsis*, <10% of genes have been linked to specific phenotypes (Lloyd and Meinke 2012); likewise, in rice, ∼10% of gene models are associated with phenotypes (Huang et al. 2022), and in maize, this is <1% (Schnable and Freeling 2011). The situation in other plants is even worse (Oellrich et al. 2015). This is not unique to plants; even in the human genome, many annotated genes have not been the subject of detailed investigation by even a single study (Sinha et al. 2018), including hundreds of genes known to be essential for cell survival (Wang et al. 2015). Research continues to focus on the same well-studied genes (Kustatscher et al. 2022a,b), thereby hindering our understanding of the core functionalities of eukaryotes and the mechanisms responsible for lineage-specific processes and phenotypes.

The process of functionally characterizing less-studied gene models or generating new hypotheses about gene–phenotype associations is time intensive and high risk. These efforts can take years and frequently yield inconclusive or null results, creating a very real career risk for researchers. These limitations constrain both reverse genetic approaches and strategies that leverage natural genetic diversity. Marker trait association signals that fall near already known genes tend to be viewed as confirmatory. In contrast, quantitative trait nucleotides located next to gene models not previously implicated in a phenotype are less pursued, largely because functional validation requires substantial time and resources and carries a high risk of yielding null results. Similar obstacles arise when interpreting results from differential gene expression analyses, coexpression networks, and other multiomic approaches. In contrast, once a gene has been shown to influence at least one phenotype, the risk of investigation declines, further amplifying attention toward genes with existing functional evidence while discouraging exploratory work on the vast number of uncharacterized gene models (Kwon et al. 2024). For example, the maize teosinte branched1 (*tb1*) gene plays an important role in reducing tillering in domesticated maize (Doebley et al. 1990, 1997). The association of *tb1* with important domestication phenotypes in maize drove the study of this gene to understand its biological role in maize and in other species (Igartua et al. 2020) with at least 140 additional studies examining *tb1* since the original characterization per the MaizeGDB (Woodhouse et al. 2025). In principle, characterization of a gene likely also reduces the perceived risk of studying other members of the same gene family, resulting on the concentration of investigation not only on specific genes but specific gene families, although the latter effect has not been rigorously evaluated in plants. Being able to predict, in advance, which genes are likely to influence phenotypes would reduce the risk of investigating currently uncharacterized or undercharacterized genes.

Previous studies have shown that classifiers can distinguish genes associated with different classes of phenotypes. For example, prior work has separated lethal from nonlethal genes in *Arabidopsis*, distinguished essential from nonessential genes across six model eukaryotes with cross-species applicability (Lloyd et al. 2015, Beder et al. 2021), classified *Arabidopsis* genes involved in primary versus specialized metabolism (Moore et al. 2019), and even predicted gene-to-phenotype associations to prioritize candidate genes for traits (Lee et al. 2010). Features used in these studies typically include both sequence-derived properties and diverse omic data collected across tissues and experiments. Beyond distinguishing biologically meaningful gene sets, previous work has also shown that annotation errors can be identified computationally, including in maize using evolutionary conservation and in *Pisum sativum* using nucleotide and amino acid composition features (Upadhyaya et al. 2022; Schulz et al. 2025). It has been shown that genes already associated with phenotypes differ from other gene models across a broad range of characteristics, and these differences appear to be consistent regardless of whether the associated phenotype is lethal, constitutive, conditional, or detectable only at the biochemical or molecular level (Schnable 2020). This pattern suggests the existence of a distinct class of gene models that either are truly unrelated to organismal phenotype or, at a minimum, are unlikely to be linked to phenotype using current experimental approaches. Studying this class of genes is challenging. The set of gene models lacking a known phenotype comprises a mixture of both genes, which could be linked to the phenotype if studied, and those truly unrelated to determining the observable phenotypes of an organism. The lack of incentives to publish null results makes it difficult to specifically identify the subset of gene models in which researchers have looked for links to phenotypes and found none. In this study, we sought to evaluate how well genes known to be associated with studied phenotypes could be distinguished from the background set of annotated gene models, whether it was possible to distinguish them based solely on features that could be derived from genomic sequence, and whether models trained in a single genetic model species are likely to perform well in other plant species.

## Results

**Figure 1.**
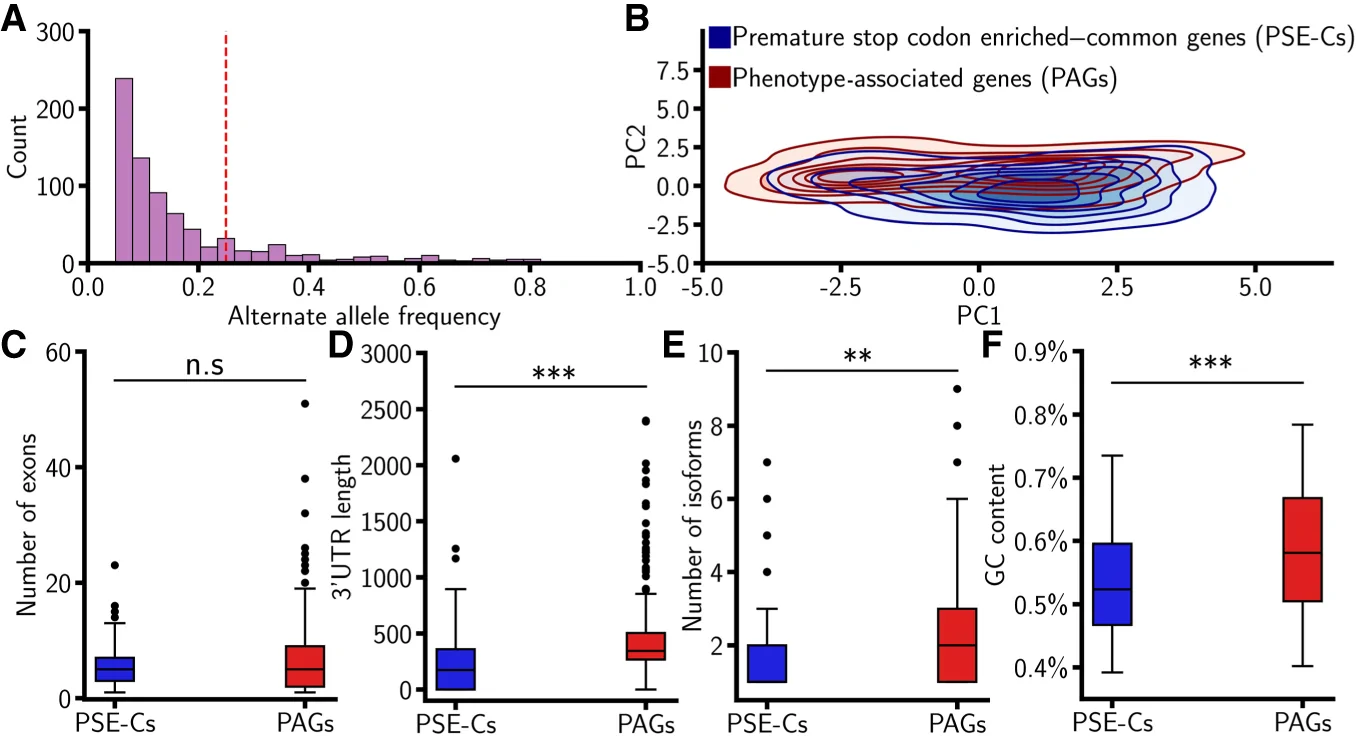
Differences between premature stop enriched–common genes (PSE-Cs) and phenotype-associated genes (PAGs). (*A*) Gene models in the B73 reference genome contain alternate alleles that introduce premature stop codons, totaling 713 models. A threshold of 0.25 alternate allele frequency defined 162 PSE-Cs, which were used as a second comparator category of gene models in this study. (*B*) Principal component analysis (PCA) of PSE-Cs (*n* = 162) and PAGs (*n* = 272). The first two principal components explain 28.62% of the variance. Data included in the PCA included a set of 2675 previously published features that were downloaded from the MaizeGDB (Sen et al. 2023). (*C*) Difference in the number of exons per gene model between PSE-Cs and PAGs. (*D*) Difference in the length of the three prime untranslated regions (3′ UTRs) of gene models between PSE-Cs and PAGs. (*E*) Difference in the number of isoforms per gene model between PSE-Cs and PAGs. (*F*) Difference in the proportion of GC content between PSE-Cs and PAGs. (**) *P*-value < 0.01, (***) *P*-value < 0.001, (n.s) nonsignificant (two-tailed Mann–Whitney *U* test).

We identified 272 maize gene models linked to phenotypic outcomes based on evidence from loss-of-function alleles and named this set as phenotype-associated genes (PAGs) (Supplemental Data Set S1). Genes for which the loss-of-function alleles have been studied and were not linked to observed phenotypic consequences were poorly documented, and it was not possible to identify a large enough set to employ as a negative set for model training. As an alternative approach to identifying genes in which loss-of-function alleles were tolerated, we identified a set of gene models in which alleles creating premature stop codons were widespread using whole-genome resequencing data from 1515 maize genotypes that include open pollinated varieties and wild relatives. This resulted in a second set of 713 gene models with variants causing a premature stop codon, that is, premature stop enriched genes (PSEs). There was no overlap between the set of PAGs and the set of PSEs (*E* = five gene models, *P*-value = 0.017, Fisher's exact test). In many cases, the alleles that produced premature stop codons were relatively rare, defined as premature stop enriched–rare genes (PSE-Rs), but in 162 cases, the frequency of the allele that resulted in a premature stop codon was greater than 0.25 that excludes across the resequenced population used to discover these variants (Fig. 1A). We focused on these 162 genes that carry common premature stop codon variants in downstream analyses, as rarer premature stop codons may be more likely to represent alleles that would impact fitness when homozygous but are still represented in our population data sets via data from open pollinated varieties or wild relatives. These gene models were supported by transcriptional data and, frequently, by proteomic and evolutionary evidence and thus do not simply represent artifacts produced by ab initio gene annotation software. Below, we refer to this set of gene models as premature stop enriched–common genes (PSE-Cs). Both PAGs and PSE-Cs were depleted in the category of gene models lacking evidence of expression in any tissue. Of the 1215 gene models meeting these criteria, three were PAGs (eight expected), and zero were PSE-Cs (five expected).

We compared PAGs and PSE-Cs across 2604 features including amino acid and nucleotide composition (as well as dinucleotide frequency, etc.), structural characterizations of gene models (e.g., sequence length, number of exons), predicted protein functional characteristics (e.g., hydrophobicity), gene expression and protein abundance data, and features derived from chromatin, coexpression, sequence variation, or transcription factor binding data sets (see Methods) (Supplemental Data Set S2). Both PAGs and PSE-Cs were less common among nonexpressed genes than among expressed genes (three nonexpressed PAGs, eight expected; zero nonexpressed PSE-Cs, five expected). PAGs and PSE-Cs exhibited statistically significant differences for ∼44% of the time (*P*-value < 0.05, two-tailed Mann–Whitney *U* test) (Supplemental Data Set S2), and 16% of these differences exceeded a more stringent Bonferroni-corrected *P*-value (*P*-value < 0.05/2604 tested features). PAGs and PSE-Cs exhibited significant differences in gene length, average protein abundance, the number of tissues expressing a gene model (Supplemental Fig. S1), and syntenic conservation in sorghum (*P*-value < 0.001, 2.2 × 10^−16^, Fisher's exact test). These results are consistent with previously reported differences between genes associated with mutant phenotypes and the overall set of annotated gene models (Schnable 2020) and also between lethal and nonlethal phenotype-associated *Arabidopsis* genes (Lloyd et al. 2015). No significant difference was observed between the number of exons in PAGs and PSE-Cs, another trait previously reported to differ between genes associated with mutant phenotypes and other annotated gene models (*P*-value = 0.53, two-tailed Mann–Whitney *U* test) (Fig. 1C). PAGs had significantly longer coding sequences (CDSs) compared with PSE-Cs, inconsistent with the possibility that the enrichment of premature stop codons results from a larger number of potential sites in which a mutation could introduce a stop codon. Many other features in which PAGs and PSE-Cs significantly differed had not previously been linked to the likelihood of being associated with organismal phenotypes. These include the length of the 3′ untranslated region (UTR; PAGs > PSE-Cs *P*-value < 0.001 [8.13 × 10^−15^], two-tailed Mann–Whitney *U* test, Cohen's d = 0.64) (Fig. 1D), number of isoforms (PAGs > PSE-Cs *P*-value = 0.003, two-tailed Mann–Whitney *U* test, Cohen's d = 0.23) (Fig. 1E), and GC content in the nucleotide sequence of the gene model (PAGs > PSE-Cs *P*-value < 0.001 [6.70 × 10^−5^], two-tailed Mann–Whitney *U* test, Cohen's d = 0.53) (Fig. 1F). Maize genes exhibit a bimodal distribution of GC content (Alexandrov et al. 2009). The distributions for both PAGs and PSE-Cs reflected the same bimodal distribution observed for maize genes as a whole, although PSE-Cs included a higher ratio of the lower GC content mode relative to PAGs (Supplemental Fig. S2). PAGs appeared to be more distinct from the overall population of annotated gene models compared with PSE-Cs. Significant differences between PAGs and non-PAG, non-PSE-Cs were detected for 54% of quantitative features (mean Cohen's d = 0.05; median Cohen's d = 0.01), with 19% remaining significant after Bonferroni correction (*P*-value < 0.05/2604 tested features). In contrast, significant differences between PSE-Cs and non-PAG, non-PSE-Cs were detected for only 28% of quantitative features (mean Cohen's d = 0.008; median Cohen's d = 0.002), with only 3% remaining significant after Bonferroni correction (*P*-value < 0.05/2604 tested features) (Supplemental Data Set S2). Collectively, these results demonstrate that PSE-Cs are more similar to the rest of the gene models compared with PAGs and that PAGs are significantly different from both PSE-Cs and the rest of the average gene models for both previously tested and untested features.

**Figure 2.**
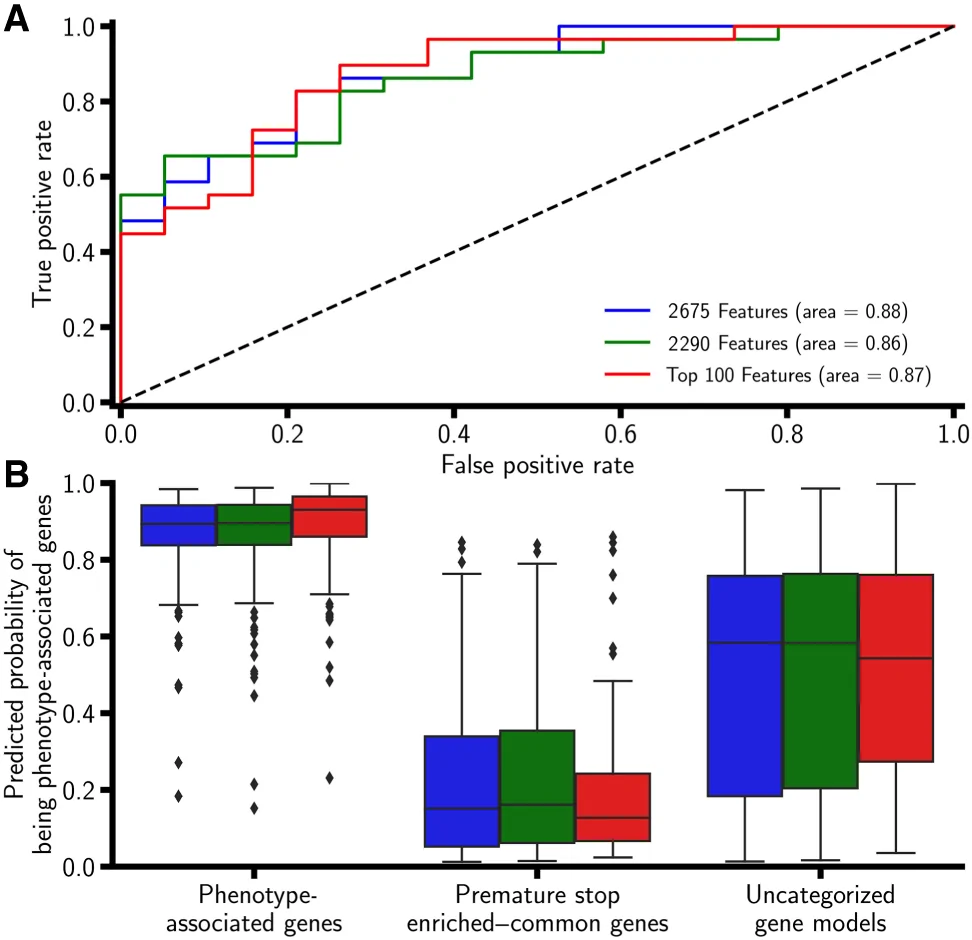
Model performance using different subsets of total features. (*A*) ROC curves are generated from three models: one using all 2675 features (nongenomic, genomic sequence–derived, and evolutionary), one using 2290 genomic sequence–derived and evolutionary features, and one using the top 100 genomic sequence–derived and evolutionary features. Performance was evaluated on 48 withheld gene models. (*B*) Predicted probability from the three models in *A* across all maize gene models.

Despite the significant differences between PAGs and PSE-Cs for many individual features, these two populations of genes exhibited only modest separation on the first principal component calculated from the full set of gene features (Fig. 1B). This indicates that there may be multiple distinct patterns of genes whose functions are likely (or unlikely) to be associated with phenotypes. This result also contrasts with the previously reported clear separation between PAGs and other annotated gene models when using a smaller, curated feature set (Schnable 2020). We trained a random forest model, which tends to be more robust to nonlinear relationships, to distinguish PAGs and PSE-Cs using all 2675 gene features (2604 continuous or numeric features + 71 binary features). Model performance was evaluated using receiver operating characteristic (ROC) curves and the area under the curve (AUC) using a holdout data set of 48 gene models (PAGs = 29, PSE-Cs = 19). ROC curves visually summarize the trade-off between true- and false-positive rates across different thresholds, whereas the ROC-AUC provides a single numeric metric that quantifies performance across all thresholds. The model exhibited strong predictive performance achieving an ROC-AUC of 0.87 on the hold-out test gene sets (Fig. 2A). The prediction probabilities assigned to PAGs (mean = 0.89) and PSE-Cs (mean = 0.17) were each significantly different from those of uncharacterized annotated gene models (mean = 0.50, *P*-value < 0.001 for both comparisons, two-tailed Mann–Whitney *U* test) (Supplemental Data Set S3). One-third of the uncharacterized maize gene models were assigned lower scores than any of the genes in our data set already linked to phenotypes, implying that a substantial fraction of annotated gene models representing sequences are unlikely to produce publishable or easy to study phenotypes (Fig. 2B). Above this threshold, many additional annotated genes were assigned scores lower than the highest-scoring PSE-C. Both of these results are consistent with a substantial fraction of annotated gene models being unlikely to produce publishable or easy to study phenotypes when disrupted. The ability of a random forest model to achieve strong predictive performance on hold-out test sets of PSE-Cs and PAGs supports the substantial differentiation of these populations of gene models.

We next sought to determine how many and which types of features were necessary to distinguish these populations of genes. The set of features describing maize gene models can be separated into genomic sequence–derived and nongenomic sequence–derived features. Genomic sequence–derived features can be directly extracted or calculated, given only an assembled genome sequence and a structural gene annotation file. In contrast, nongenomic sequence–derived features, including gene expression data, protein abundance, chromatin accessibility profiling, and assays of transcription factor binding, require additional experiments and are typically challenging to generate similarly across experiments conducted in multiple species. Among the 100 features with the highest Gini importance in the trained PAG/PSE-C classification model, 12 were derived from nongenomic sequence or expression data. Gini importance quantifies how much each feature contributes to reducing classification error across the decision trees in the model. Only one of the top 10 most important features was not derived from genomic sequence, namely, the maximum fragments per kilobase of transcript per million mapped reads (FPKM) observed for a gene across all tissue samples (Supplemental Data Set S4). Syntenic conservation of a gene model in sorghum appeared to be the most important feature (Supplemental Data Set S4). The top 100 features also included gene structural attributes, particularly 5′- and 3′-UTR lengths, as well as numerous protein descriptors reflecting amino acid composition, residue ordering, physicochemical properties, and local sequence architecture. Other prominent features included *k*-mer frequencies, frequency of occurrence of each amino acids (codon usage), the number of times a codon appears in a gene divided by the number of expected occurrences under equal codon usage (relative synonymous codon usage), and GC content at the third codon position consistent with differences between PAGs and PSE-Cs (Fig. 1F). A random forest model trained solely on genomic-derived and evolutionary features achieved comparable performance, with an ROC curve area of 0.86 (Fig. 2A,B). Model performance remained consistent with an ROC curve area of 0.87 when the feature set was restricted to only 100 genomic sequence–derived features with the highest Gini importance (Fig. 2A,B). Consistent with previous models, synteny remained the most important feature. The probability of a gene in the top 10% (threshold = 0.88, *n* = 3904) of predicted scores generated by the top 100-feature genomic sequence–derived data model being linked to a phenotype was estimated to be approximately 18.3 times higher than that of a gene with a score in the bottom 50% (threshold = 0.88, *n* = 19,516) based on evaluation of the hold-out test data. These results suggest that it may be feasible to build high-performing models based solely on feature sets that can be generated consistently across different species.

**Figure 3.**
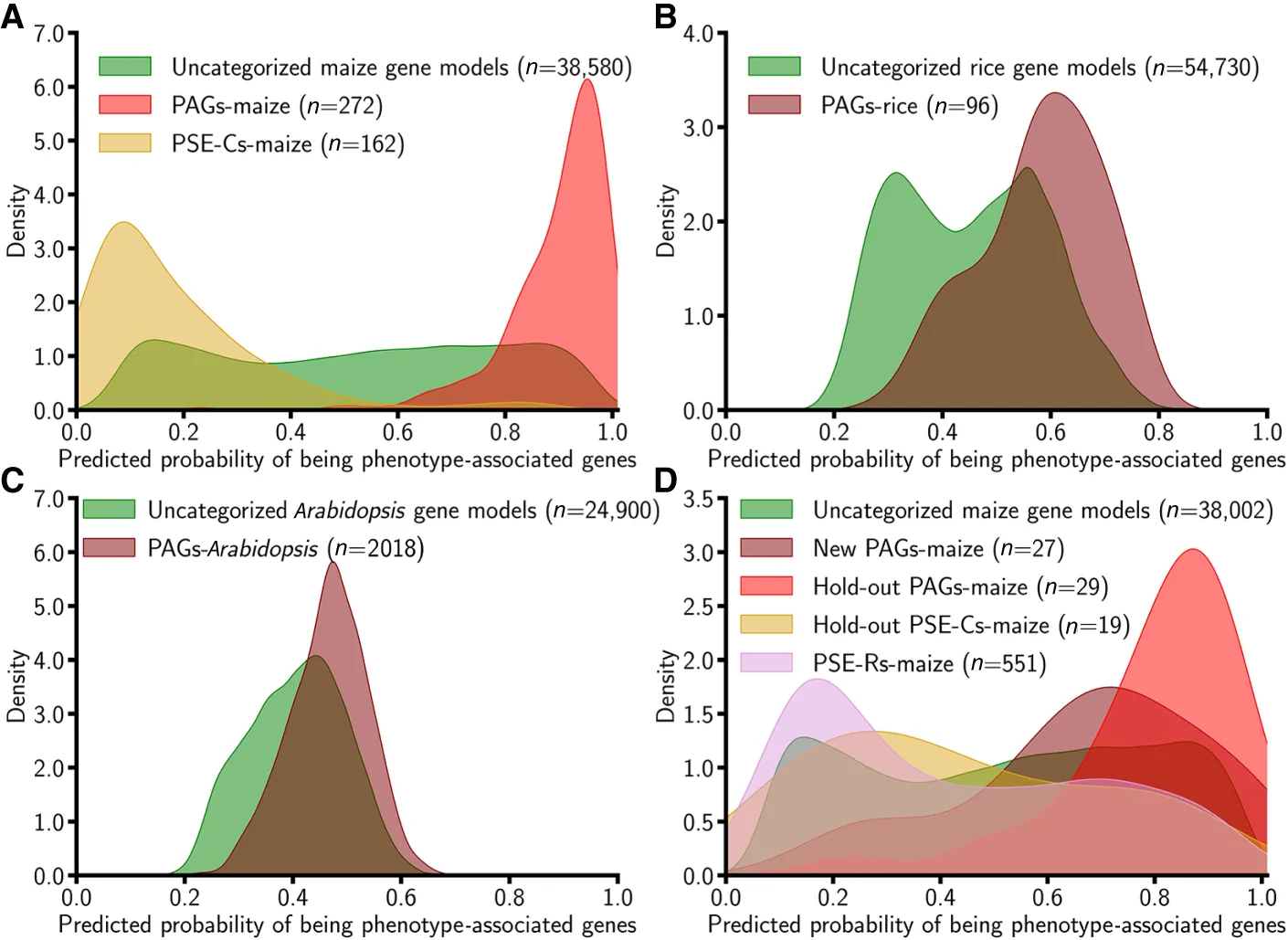
Phenotype-associated genes (PAGs) have higher predicted probabilities compared with uncategorized gene models in maize, rice, and *Arabidopsis*. (*A*) Distribution of predicted probabilities of PAGs, premature stop enriched–common gene (PSE-C) models, and uncharacterized maize gene models. (*B*) Distribution of predicted probabilities of a subset of validated gene models (*n* = 96) retrieved from the literature in rice (Huang et al. 2022) using a model trained on maize data with 99 genomic sequence–derived and evolutionary features. (*C*) Distribution of predicted probabilities of a subset of validated gene models (*n* = 2018) retrieved from the literature in *Arabidopsis* (Lloyd and Meinke 2012) using a model trained on maize data with 99 genomic sequence–derived and evolutionary features. (*D*) Predicted probability distribution of newly validated PAGs (*n* = 27, curated from the MaizeGDB since November 27, 2021) compared with hold-out PAGs, hold-out PSE-Cs, premature stop enriched–rare genes (PSE-Rs), and uncategorized gene models.

The model trained solely on genome sequence–derived and comparative genome–derived features was able to separate the prediction probability distribution of PSE-C maize gene models from PAGs in maize, with uncharacterized gene models having overlapping distribution with both extreme classes (Fig. 3A). We next tested how well our model translates to other plant species. The genetic characterization of several plant species, notably rice and *Arabidopsis*, also includes information on the phenotypic consequences of disrupting comparable or greater numbers of genes compared with the maize data set used in this study. However, in most plant species, with sequenced genomes, few or no genes have been directly linked to mutant phenotypes. We assessed the transferability of a model trained on maize data to other species using a model trained on 99 of the top 100 maize features, excluding one feature that could not be easily generated for rice gene models (see Methods), to predict the probability that annotated rice genes would be associated with phenotypic consequences. From the set of approximately 4500 cloned rice genes in the funRiceGenes database (https://funricegenes.github.io/; Huang et al. 2022), we manually curated a nonexhaustive set of 100 that met the same phenotype criteria applied to maize. The 96 rice PAGs that could be mapped to MSU v7 gene models included 72 that were conserved at syntenic locations in maize and 24 that lacked maize syntenic orthologs. However, in only seven cases was the maize syntenic ortholog of a rice PAG also classified as a PAG in maize. The 96 rice PAGs were assigned significantly higher probabilities of being associated with a phenotype than with the background set of annotated gene models in the rice genome (*P*-value < 0.001 [5.27 × 10^−19^], two-tailed Mann–Whitney *U* test) (Fig. 3B; Supplemental Data Set S5). The probability of a rice gene in the top 10% (threshold = 0.63, *n* = 5564) of scores being linked to a phenotype was 11 times higher than that of a gene in the bottom 50% (threshold=0.45, *n* = 27,015). The divergence of the lineages leading to maize and rice is estimated to have occurred ∼50 million years ago, roughly one-third the time since the estimated age of the most recent common ancestor of maize and *Arabidopsis* (∼160 million years ago) (Kumar et al. 2017). The *Arabidopsis* genome is also <10% the size of the maize genome, with substantially lower transposon content. A model trained solely on maize data using the same 99 features assigned significantly higher probabilities of being associated with a phenotype to a set of *Arabidopsis* PAGs than to the background set of annotated gene models in the *Arabidopsis* genome (*P*-value < 0.001 [1.12 × 10^−176^], two-tailed Mann–Whitney *U* test) (Fig. 3C; Supplemental Data Set S5). The prediction probability was not different between essential, morphological, cellular, and biochemical *Arabidopsis* PAGs and conditional *Arabidopsis* PAGs (*P*-value = 0.77, two-tailed Mann–Whitney *U* test) (Supplemental Fig. S3). The probability of an *Arabidopsis* gene model in the top 10% (threshold = 0.52, *n* = 2806) of scores being linked to a phenotype was four times higher than a gene in the bottom 50% (threshold = 0.41, *n* = 13,227). These results confirm the notion that a model trained solely on maize data can still achieve both statistically significant and operationally meaningful performance in prioritizing genes for phenotypic characterization when applied to a distantly related angiosperm with a radically different genome architecture.

Cross-validation and testing on a randomly selected set of hold-out test data are both widely used approaches to estimate the performance of machine learning models. However, the accuracy of machine learning models can sometimes decline when evaluated on truly out of sample data sets relative to estimates based on held-out subsets of the same data set used to train the initial model (Guan et al. 2025; Lei et al. 2025). We generated a new, curated set of maize PAGs from literature published after our original PAG data set was finalized (Supplemental Data Set S7). The estimated probabilities of being linked to a phenotype of these new out-of-sample PAGs (*n* = 27) were significantly higher than those of a typical uncharacterized gene (*P*-value = 0.002, two-tailed Mann–Whitney *U* test) (Fig. 3D). The average estimated probability assigned to the out of sample PAG genes was modestly lower than the probabilities assigned to PAGs from the original hold-out test data set (*µ* = 0.68 vs. *µ* = 0.79). However, the estimated probability that a gene in the top 10% of the prediction scores is associated with a phenotype was still approximately 13 times as high as the probability for a gene in the bottom 50% of scores. This ratio was estimated to be 18.3 times using the original hold-out test genes. These results demonstrate that the prediction model retains strong predictive performance even when applied to truly out-of-sample data sets.

**Figure 4.**
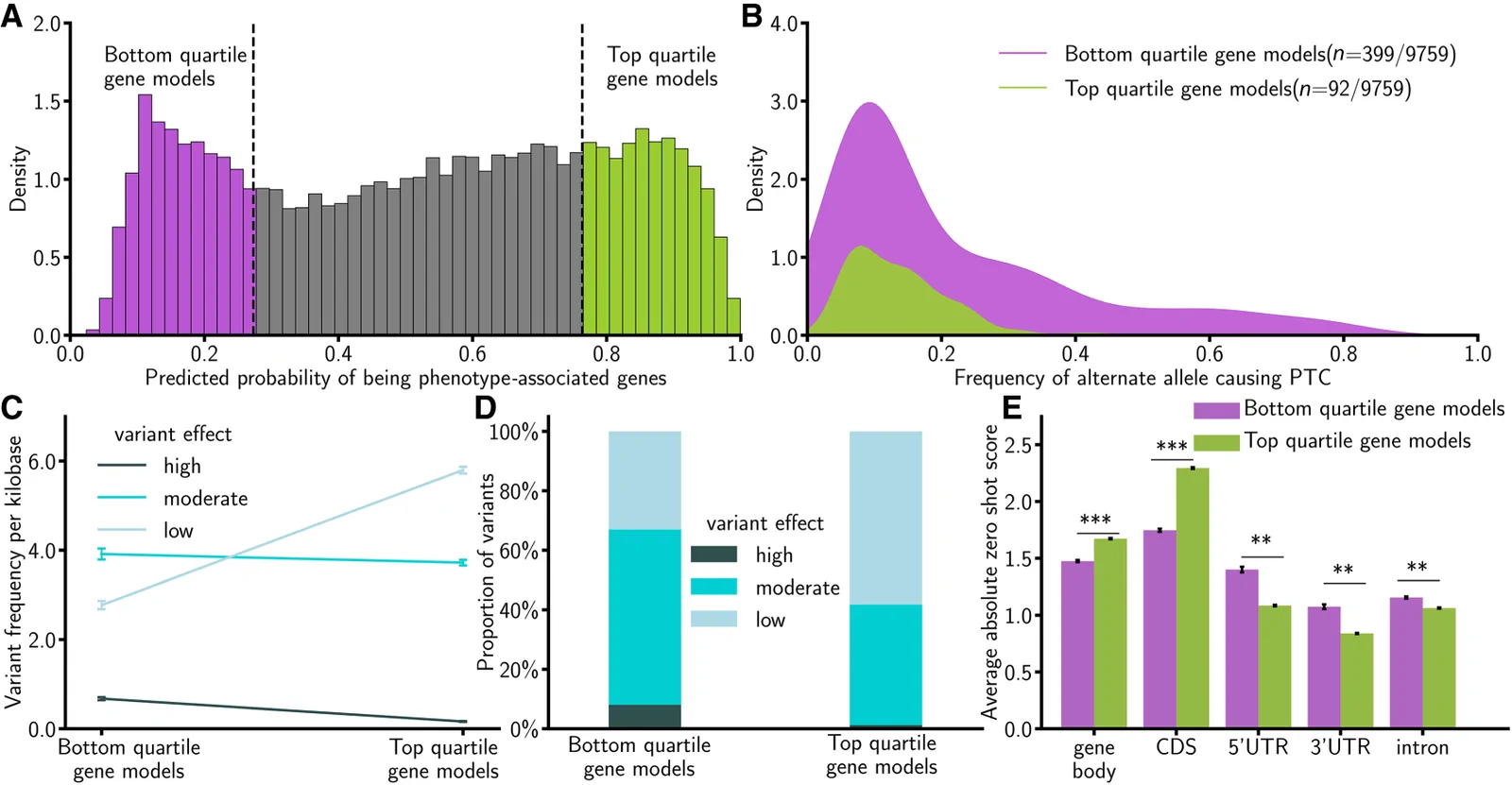
Genes with higher estimated effects on plant phenotypes are conserved in the maize population. (*A*) Bottom- and top-quartile gene models were defined from the predicted probability distribution derived from the model trained with the top 100 genomic sequence–derived and evolutionary features. (*B*) The bottom-quartile gene models show approximately 4.4-fold higher odds of containing premature stop codons compared with the top-quartile models (*P*-value < 2.26 × 10^−16^, Fisher's exact test). (*C*) Variant frequency per kilobase in coding sequences of the primary transcript of the gene models, separated by effect type. Bottom-quartile models have higher frequencies of high-effect (*P*-value = 9.03 × 10^−33^) and moderate-effect variants (*P*-value = 0), whereas top-quartile models have higher frequencies of low-effect variants (*P*-value = 0; two-tailed Mann–Whitney *U* test). (*D*) Proportional distribution of high-, moderate-, and low-effect variants between quartiles. (*E*) Average absolute zero-shot score for variants estimated from a pretrained DNA language model, PlantCaduceus (Zhai et al. 2025), in different parts of the gene models between the bottom-quartile gene models and top-quartile gene models from *A*. (**) 0.01 < *P*-value < 1 × 10^−20^, (***) *P*-value < 1 × 10^−200^ (two-tailed Mann–Whitney *U* test).

The gene models predicted to be linked to phenotypes are supported by other data types associated with purifying selection or the consequences of mutations. Gene models in the bottom quartile (threshold = 0.27, *n* = 9759) of the probability of being associated with a phenotype exhibited approximately 4.4 times higher odds of containing a premature stop codon compared with those in the top quartile (threshold = 0.76, *n* = 9759, *P*-value < 2.2 × 10^−16^, Fisher's exact test) (Fig. 4A,B). Variants introducing premature stop codons in bottom-quartile gene models were observed at higher minor allele frequencies than were variants introducing premature stop codons in top-quartile genes (*P*-value = 0.01, two-tailed Mann–Whitney *U* test). Based on alignments to orthologous genes in sorghum, bottom-quartile genes exhibited higher rates of sequence evolution at both the DNA sequence and protein sequence level with elevated rates of synonymous substitutions (median *K*_s_, 0.53 vs. 0.28, *P*-value < 0.001), elevated nonsynonymous substitutions (median *K*_a_, 0.19 vs. 0.03, *P*-value < 0.001), and elevated *K*_a_/*K*_s_ (median *K*_a_/*K*_s_, 0.14 vs. 0.20, *P*-value < 0.001), consistent with relaxed purifying selection or a mixture of purifying and positive selection. Bottom-quartile gene models showed higher CG and CHG methylation levels across annotated gene regions compared with top-quartile gene models. In contrast, CHH methylation across annotated gene regions was higher in top-quartile gene models than in bottom-quartile gene models, consistent with the bottom-quartile set not being primarily composed of transposon fragments. The variant frequency per kilobase of CDS was higher in top-quartile gene models than in bottom-quartile gene models, but this effect was driven by a higher prevalence of variants predicted to have small (low) effects on protein function, typically synonymous variants, and the proportion of variants predicted to have moderate (*P*-value < 0.001, two-tailed Mann–Whitney *U* test) or strong (high) effects on protein function (*P*-value < 0.001, two-tailed Mann–Whitney *U* test) were significantly higher in bottom-quartile gene models (Fig. 4C,D). The apparent inconsistency between higher *K*_a_ and *K*_s_ ratios both being observed in bottom-quartile genes and a higher overall variant frequency being observed in top-quartile genes may have resulted from the subset of genes that lacked syntenic orthologs in sorghum. These genes were still included in estimates of variant frequency, but it was not possible to estimate substitution rates for these genes. A similar pattern may also arise because bottom-quartile genes were more likely to show presence–absence variation. When a gene is present in some maize lines but absent in others, SNP and indel calls within that gene tend to have higher levels of missing data and are therefore more likely to be removed during quality filtering. We employed PlantCaduceus, a DNA language model that has learned evolutionary conservation patterns in angiosperm genomes (Zhai et al. 2025), to estimate the effects of DNA substitutions without the need to align against genes in related species. Genetic variants in the gene body or CDS of top-quartile gene models exhibited significantly higher predicted likelihoods of being deleterious (based on average absolute zero-shot scores from PlantCaduceus) compared with bottom-quartile gene models (*P*-value < 1 × 10^−200^, two-tailed Mann–Whitney *U* test) (Fig. 4E). In contrast, variants present in UTRs or introns exhibited a small but significant bias in the opposite direction, with variants in these regions of bottom-quartile genes assigned marginally higher absolute zero-shot scores by PlantCaduceus compared with those in top-quartile genes (*P*-value < 1 × 10^−20^, two-tailed Mann–Whitney *U* test) (Fig. 4E).

To assess whether the model was prioritizing members of well-annotated gene families, we examined Gene Ontology (GO) term enrichment analysis among genes from the top quartile and bottom quartile of the distribution, constraining GO enrichment analysis to the population of only those genes assigned at least one GO term. At an average, top-quartile gene models were assigned 17 GO term per gene model, and bottom-quartile gene models were assigned six GO terms per gene model (minimum GO term assigned to a gene model: one; maximum GO term assigned to a gene model: 297) (Supplemental Fig. S4). GO enrichment analysis showed that top-quartile genes were enriched in 424 GO terms (out of 7277 GO terms tests), and bottom-quartile genes were enriched in 37 GO terms (Supplemental Data Set S6). This result raised the concern that the model's strong performance might partly reflect memorization of the characteristics of well-annotated gene families. Ninety-six percent of top-quartile genes and 47% of bottom-quartile genes were assigned at least one GO term. However, 157 genes in the top decile of predicted probabilities of being phenotype-associated encode proteins with domains of unknown function. Manual curation of the top 15 uncharacterized gene models showed that four genes had only domains of unknown function. These results indicate that the model captured biologically important patterns to distinguish gene models highly likely to be associated with phenotype and that genes predicted to be associated with phenotypes extend beyond those of well-characterized gene families.

## Discussion

A substantial proportion of gene models remain uncharacterized (Peña-Castillo and Hughes 2007; Schnable and Freeling 2011; Stoeger et al. 2018). Importantly, this lack of functional characterization is not limited to individual gene models, as some entire gene families also remain poorly understood. The time and resources required to functionally characterize an individual gene remain costly. As a result, the social structure of research and funding allocation decisions incentivize focusing on well-studied genes. Therefore, more than three-fourths of gene models across humans and plants are understudied and have not been functionally characterized (Stoeger et al. 2018; Schnable 2020). Although the initial impulse may be to assume that all currently uncharacterized gene models are equally good candidates for characterization, here, we demonstrated that it is possible to train models on relatively simple and generalizable sets of features that increase the odds of successful reverse genetic characterization. We curated an extended list of PAGs identified in maize from the 1900s to November 27, 2021, and identified a complementary set of PSE-C models that can serve as a negative set for model training. All of the genes in this set had evidence of transcription (e.g., expression in RNA-seq studies); many had evidence of translation from proteomic investigations; and more than one-third were conserved at syntenic locations in the sorghum genome, indicating these loss-of-function tolerant genes did not simply represent artifacts produced by ab initio gene annotation software. Consistent with this, even after restricting the analysis to gene models classified as true genes by reelGeneScore (threshold > 0.5, removing more than one-fourth of maize gene models) (Schulz et al. 2025), the evolutionarily informed model still distinguished PAGs with an ROC-AUC of 0.78. This is a decline relative to the ROC-AUC of 0.87 achieved when all annotated genes were included but is still much higher than the 0.5 expected for an uninformative model. Together, these findings support that the predictions generated by the model represent biologically meaningful differences rather than solely the identification of annotation artifacts. The difference between PAGs and PSE-Cs could not be explained by a difference in the gene family size to which these gene models belong (*P*-value = 0.13, two-tailed Mann–Whitney *U* test). This was previously found to be an important difference between genes associated with lethal phenotypes and genes associated with nonlethal phenotypes, in which presence of even a single paralog reduces the likelihood of lethality under disruption of a gene (Lloyd et al. 2015). A simple classifier model provided with evolutionary information and genomic sequence–derived features was able to classify gene models in maize into the correct classes with an ROC-AUC of 0.87.

The top quartile of genes predicted to be linked to phenotypes included >20% of all maize gene models encoding proteins containing domains of unknown function; more than 300 genes in the top quartile lacked GO annotations; and more than 200 genes lacked any functional description at all, including automated domain annotations. This suggests that our model is not simply learning the properties of well-characterized gene families as uncharacterized genes and that members of uncharacterized gene families were not excluded from those predicted to be associated with phenotypes. Syntenic conservation in a closely related species (specifically sorghum for the maize model) consistently ranked as the most important across all trained models. However, when nonsyntenic genes were excluded from both the training and testing set (removing the information content of synteny), a retrained top-100-feature model was able to classify maize gene models with an ROC-AUC of 0.85 compared with 0.87 for the original model, indicating that although synteny is an important predictor, its inclusion is not essential to achieve substantial prediction performance.

PAGs were defined based on reports in the literature describing visible phenotypes in relatively easy-to-characterize tissues. This definition necessarily biased our training data set against genes whose phenotypes are most apparent in tissues or organs that are more difficult to observe, such as roots. Our initial maize PAG set was also enriched for genes associated with easily characterized kernel phenotypes. Of the 27 newly validated gene models, nearly one-half were kernel mutant phenotypes. However, kernel-related and non-kernel-related genes showed no significant difference in mean predicted probability of being phenotype-associated (*P*-value = 0.39, two-tailed Mann–Whitney *U* test), and in fact, the newly characterized genes with non-kernel-related phenotypes were assigned higher mean probabilities by the model (mean = 0.63 vs. mean = 0.72), suggesting the high representation of these genes did not bias the model toward genes with phenotypes observed specifically in kernels. Our criteria for the training data set also specifically excluded genes linked to phenotypes only under specific environmental conditions (e.g., stress specific phenotypes). However, *Arabidopsis* genes associated with conditional phenotypes were not assigned significantly lower predicted probabilities of being phenotype-associated compared with *Arabidopsis* genes associated with constitutive phenotypes (Supplemental Fig. S3).

We were able to assess overfitting or species-specific prediction by testing models trained in maize on known PAGs from other species. Models trained in maize generalized well across species, assigning substantially higher predicted phenotype probabilities to genes with known mutant phenotypes in both rice and *Arabidopsis*, two distantly related species with large differences in genome size and gene content. Using a top-decile threshold yields very low false-positive rates but misses many true PAGs, indicating a substantial false-negative rate. In the absence of confirmed true-negative gene set, predicted probabilities should be interpreted as relative prioritization scores rather than as absolute probabilities of phenotype association. It should also be noted that the probability threshold corresponding to any given percentile of scores varied across the species we evaluated. Application of the model may be more comparable when applying similar percentile based threshold compared with comparable absolute scores. Selection of specific threshold would necessarily be dependent on a user's relative tolerance for false positives and false negatives. As additional genes continue to be cloned and functionally characterized, it will be informative to reassess model performance and determine how well the current patterns hold in larger and more diverse validation sets.

One potential application of our classifier is predicting the likelihood that disrupting a given gene will be associated with phenotypic changes. Association studies with genetic markers narrow down the genomic region associated with a given trait of interest. There are cases in which the causal gene is the nearest one to the associated genetic marker (Sahay et al. 2023), especially when there is a high-density genetic marker across the genome and strong linkage disequilibrium decay (Grzybowski et al. 2023). However, for many plants, markers may be at low density or there may be strong linkage disequilibrium or, in some cases, a combination of both. Having a complementary tool that provides a quantitative score for each gene model indicating the probability that a gene model is linked to a phenotype could guide researchers in prioritizing candidate genes in such association studies. Such a framework could help reduce uncertainty when large genomic intervals contain dozens or even hundreds of annotated gene models. In addition, this tool could provide complementary evidence to traditional QTL mapping or GWAS approaches, ultimately accelerating functional validation. The vast majority of gene models, even in the most studied plants such as maize, rice, and *Arabidopsis*, are yet to be characterized, let alone many orphan crops, which are equally, if not more, important (Shrestha et al. 2023). Editing, validating, and characterizing gene models takes a long time and is costly (Stoeger et al. 2018). Quantitative scores for gene models that could be used as confidence scores for prioritizing targets would therefore be invaluable. Such scores could help researchers focus their limited resources on the most promising candidates, thereby accelerating basic discovery and crop improvement. Identifying true genes based on high quantitative scores could mark the starting point for optimizing their functional roles across diverse crop species.

## Methods

### Definitions of PAG and PSE sets

The initial set of 272 maize genes whose loss of functional alleles has been experimentally shown to produce a detectable change as an easily observable phenotype, along with the organ affected by each gene, was defined through our manual curation of a larger set of 603 maize locus records annotated as phenotype-associated and retrieved from the MaizeGDB (Sen et al. 2023) on November 27, 2021. This was done to exclude loci linked to purely molecular phenotypes (e.g., variation in gene expression) or when the evidence for a genotype–phenotype link came from studies using techniques with higher false-positive rates (e.g., association studies). From the set of approximately 4500 cloned rice genes in the funRiceGenes database, we manually curated a nonexhaustive set of 100, which met the same phenotype criteria applied to maize (Huang et al. 2022). In four cases, a gene identified in the analysis did not correspond to a gene in the sequence-derived feature set calculated from the MSU v7 rice genome annotations, leaving 96 phenotype-associated rice genes. The set of PAGs in *Arabidopsis* was retrieved from a previously published list (Lloyd and Meinke 2012). Excluding gene models in the Lloyd and Meinke list with phenotypes that were classified as “NC; nonconfirmed” resulted in a set of 2018 PAGs for *Arabidopsis*. Out-of-sample maize PAGs were identified using a set of 2582 citations (through April 14, 2025) describing maize loci that had not been associated with a phenotype in our original November 27, 2021, data set. These citations were derived from 682 unique papers, and screening these publications identified an additional 27 loci that met the criteria for publishing a new genotype-to-phenotype association supported by loss-of-function data.

The maize PSE set was defined using a published set of genotype calls derived from whole-genome resequencing of 1515 maize varieties (Supplemental Data Set S8; Grzybowski et al. 2023). The functional consequence of each of the approximately 46 million biallelic genetic variants (SNPs and indels) identified within this study were annotated using SNPeff v5.1d (De Baets et al. 2012) and the published gene model annotations of the B73_RefGen_V5 reference genome (Hufford et al. 2021). The primary transcript of each annotated gene model containing a genetic variant that produces a premature stop codon and is present at an alternate allele frequency greater than 0.25 across the population was considered a PSE-C, resulting in a final data set of 162 PSE-C maize genes.

### Definition of the feature data set describing maize gene models

A data set of 14,418 different features describing 39,756 annotated gene models in the B73_RefGen_V5 reference genome was downloaded from the Maize Feature Store in the MaizeGDB (Sen et al. 2023, https://mfs.maizegdb.org/featurebase). Gene models that were not assigned to any of the 10 maize chromosomes (i.e., on unanchored scaffolds) were dropped from the data set, reducing the total number of gene models to 39,034. Redundant features introduced by the presence of both raw and normalized values in the data set, features with missing values for more than one gene model, annotation of gene model in sorghum version 3.1 annotation and in Tzi8, chromosome assignment, and four subcellular localization features while retaining only one subcellular localization feature, Subcel1, were removed from the data set. In addition, features describing tripeptide amino acid composition, which would otherwise have constituted ∼55% of the total feature set, were also dropped from the data set. Gene expression and protein abundance values of “none” were converted to “zero” prior to analysis. After excluding these features, 2639 remained, including features describing gene structure, codon usage, nucleotide and protein composition, and nongenomic sequence–derived data regarding gene expression atlas, protein expression, chromatin-related features, expression localization, and transcription factor binding sites associated with each gene model in maize. A single non-MaizeGDB-sourced feature, syntenic conservation in sorghum, scored using the data set published by Dias et al. (2026), was added, resulting in a final data set of 2640 unique features. Synteny based on sorghum gene models included a filtering step that removed members of tandem duplicate arrays that did not possess reciprocal best BLAST hit relationships between maize and sorghum. The feature generation process and definition of features have been described in detail by Sen et al. (2023). The final feature set of 2640 features included 31 binary features, five categorical features, and 2604 continuous and discrete features. A total of 350 features were nongenomic sequence derived, while the remaining 2290 features could be directly computed from the maize genome assembly and annotation files.

For use in machine learning applications, categorical features were one-hot-encoded, increasing the effective number of variables to 2675 (Supplemental Data Set S9), and continuous and discrete features were min-max-normalized across all gene models using the MinMaxScaler function in scikit-learn v1.3.0 implemented in Python v3.11.4 (Pedregosa et al. 2011).

### Random forest classifier model for prediction

Before developing or conducting any analysis, ∼10% of the gene models were set aside as a separate testing data set. The data were split into training and testing data sets using a previously proposed family-guided strategy (Washburn et al. 2019) and gene family assignments retrieved from PLAZA (Van Bel et al. 2022) to minimize the risk of data leakage. The testing data set included 29 PAGs and 19 PSE-Cs. For each model trained in this study, the remaining 386 labeled gene models were used to optimize the hyperparameters and train the final models. The number of trees in a model, number of features for splitting each tree, maximum depth of the tree, minimum number of samples required to split a node, minimum number of samples required to be at a leaf node, and whether bootstrap samples were used when building trees were optimized via threefold cross-validation of the training data set using the RandomizedSearchCV function in Scikit-learn v1.3.0 implemented in Python (Pedregosa et al. 2011). The optimal set of hyperparameters identified through this process for each model was then used to train the respective final model using the whole training data. ROC curves were plotted based on the model's performance on the hold-out test data set, and the AUC was used to evaluate the model's performance.

Feature importance scores for both models using 2675 and 2290 features were calculated using the built-in feature_importance_ function of a trained random forest model from the scikit-learn library implemented in Python. This method estimates the importance of each feature based on the mean decrease in impurity across all trees in the ensemble. We were only able to generate values for 99 of the 100 maize features of greatest estimated importance in rice and *Arabidopsis*. One feature, “gene age” was not included in cross-species prediction. A new model trained on maize data using only those 99 genomic features and the same procedure described above was employed for cross-species predictions.

### Validation of model predictions

Variant density was calculated by dividing the number of variants with minor allele frequency greater than 0.05 from the same resequencing-based data set used to identify PSE occurring in the CDS of the primary transcript by the CDS length of the primary transcript of that same gene. Additional density values were generated by classifying genetic variants present within the primary transcript of a given gene into low-effect, moderate-effect, modifier-effect, and high-effect categories via SnpEff (De Baets et al. 2012). CHH methylation per gene model, rates of synonymous (*K*_s_) and nonsynonymous substitutions (*K*_a_) based on alignment to orthologous genes in sorghum, and *K*_a_/*K*_s_ data were downloaded from the MaizeGDB webpage (Woodhouse et al. 2025, https://mfs.maizegdb.org/Other, https://mfs.maizegdb.org/DNAmethylation). The functional impact of genetic markers present between the annotated start and stop sites of each gene of interest was estimated using zero-shot scores provided by PlantCaduceus (Zhai et al. 2025). The absolute value of the log-likelihood difference between reference and alternate alleles was used when the reference or the alternate allele was assigned low relative probabilities, both of which indicate the presence of functional differences segregating in the population. ReelGene scores were downloaded for version 4 maize gene models (Schulz et al. 2025), which were then mapped to version 5 maize gene models. Gene models from version 5 genome annotation that mapped to multiple version 4 gene IDs were excluded, resulting in the removal of ∼25% of gene models in version 5, which was likely owing to gene annotation error in version 4 but was resolved in version 5. InterProScan V5.75 was used to identify gene models whose translated CDS coded for one or more domains of unknown function (Jones et al. 2014). Tests for GO enrichment or purification were performed using the Python library GOATOOLS v1.4.12 (Klopfenstein et al. 2018). The GO annotation file “Zm00001eb.1.fulldata.txt.gz” for B73 reference genome version 5 was downloaded from the MaizeGDB (Hufford et al. 2021). To exclude bias introduced by the presence of greater proportions of some gene sets representing entirely uncharacterized proteins, the background set of all analyses was defined as the set of genes associated with one or more GO terms unless otherwise specified.

### Curation of rice and *Arabidopsis* gene model features

The rice genome was retrieved from the Rice Genome Annotation Project (Kawahara et al. 2013; Hamilton et al. 2025), and the *Arabidopsis* genome was retrieved from The *Arabidopsis* Information Resource (TAIR) (Lamesch et al. 2012). For both species, only data from primary transcripts were used, and genes annotated as transposable elements (TEs) were excluded. Length features including 3′-UTR length, 5′-UTR length, total exon length, and average exon length were directly calculated from gene annotations in the GFF3 file. *k*-mer ratios were derived using a custom R script that integrated the FASTA sequence with GFF3 annotations. Codon usage frequency, relative synonymous codon usage (RSCU), and GC content at the third codon position (GC3s) were also computed from representative CDSs.

Multiple protein sequence descriptors were extracted from one representative peptide sequence per gene using the protr R package (Xiao et al. 2015; R Core Team 2021). After removing stop codons and filtering out sequences shorter than three amino acids, the following descriptor types were computed: triad composition; Geary, Moran, and Moreau–Broto autocorrelations, dipeptide composition; pseudo–amino acid composition (PAAC); quasi-sequence order (QSO); and composition–transition–distribution (CTD: CTDC, CTDD, CTDT). Custom functions with built-in error handling were applied to extract specific subsets of features.

All computations were implemented in parallel to accelerate processing. Syntenic rice genes were identified based on orthology with sorghum genes and directly obtained from the Supplementary Materials of a previously published study (Dias et al. 2026). Syntenic *Arabidopsis* genes were inferred via comparison with *Brassica rapa* genes using the latest reference genome and the SynOrths pipeline (Cheng et al. 2012). A single feature “gene age” was not included, as no reliable public resources were found, and they could not be readily derived from sequence or annotation data using straightforward computational methods.

### Code availability

All the code used to train, test, and evaluate the model, along with the models trained in this study, is available at GitHub (https://github.com/NikeeShrestha/GenePrediction_Paper) and as Supplemental Code.

## Supplemental Material

Supplement 1

Supplement 2

Supplement 3

Supplement 4

Supplement 5

Supplement 6

Supplement 7

Supplement 8

Supplement 9

Supplement 10

Supplement 11
