# Supplementary material for: A sequence-based classifier distinguishes phenotype-associated genes from other gene models in plants
Source: Genome Res. 2026 Sep;36(9):1863–72. doi: 10.1101/gr.281802.125 (PMC13533879; doi:10.1101/gr.281802.125)
Supplement: Supplementary file 1 [file Supplemental_Figures.docx]

Supplemental Figure S1: Difference in a few genomic and non-genomic sequence-derived features between PSE-Cs and PAGs. A). Difference in gene length between PSE-Cs and PAGs. B). Difference in the number of tissues expressing a gene model between PSE-Cs and PAGs. C). Difference in the average protein abundance between PSE-Cs and PAGs. ** p-value < 0.01, *** p-value < 0.001 (two-tailed Mann-Whitney *U* test).

Supplemental Figure S2: Frequency of guanine (G) and cytosine (C) in phenotype-associated genes (PAGs), premature stop-enriched common genes (PSE-Cs) and uncharacterized gene models.

Supplemental Figure S3: Distribution of prediction probability of different classes of phenotype-associated genes in *Arabidopsis* (Lloyd et al., 2012) compared with uncharacterized *Arabidopsis* gene models.

Supplemental Figure S4: Distribution of the number of Gene Ontology (GO) terms associated across maize gene models. A total of 7,277 GO terms were assigned to 30,395 maize gene models. Seventy-eight maize gene models that were assigned more than 100 GO terms are excluded from this figure to improve visualization.
